# (*E*)-2-{*N*-Ethyl-4-[(4-nitro­phen­yl)diazen­yl]anilino}ethyl acrylate

**DOI:** 10.1107/S1600536808003085

**Published:** 2008-04-02

**Authors:** Mohammad Yousefi, Hossein Hosseini, Vahid Amani, Mansour Arab Chamjangali, Hamid Reza Khavasi

**Affiliations:** a Islamic Azad University, Shahr-e-Rey Branch, Tehran, Iran; b Department of Chemistry, Faculty of Science and Engineering, Imam Hossein University, Tehran, Iran; c Department of Chemistry, Shahrood University of Technology, Shahrood, Iran; d Department of Chemistry, Shahid Beheshti University, Tehran 1983963113, Iran

## Abstract

In the mol­ecule of the title compound, C_19_H_20_N_4_O_4_, the rings are almost coplanar, forming a dihedral angle of 0.76 (3)°. In the crystal structure, inter­molecular C—H⋯O hydrogen bonds link the mol­ecules.

## Related literature

For related literature, see: Peters & Freeman (1991 ▶); Gregory (1991 ▶); Gur *et al.* (2007 ▶); Venkataraman (1970 ▶); Srinivasa *et al.* (2003 ▶). For bond-length data, see: Lacroix *et al.* (2000 ▶); Gunnlaugsson *et al.* (2001 ▶).
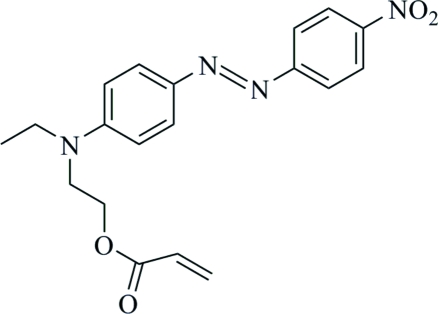


## Experimental

### 

#### Crystal data

C_19_H_20_N_4_O_4_
*M* *_r_* = 368.39
Orthorhombic, 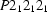

*a* = 8.1518 (9) Å
*b* = 10.6651 (11) Å
*c* = 20.6782 (19) Å
*V* = 1797.8 (3) Å^3^
*Z* = 4
Mo *K*α radiation
μ = 0.10 mm^−1^
*T* = 120 (2) K
0.5 × 0.2 × 0.06 mm

#### Data collection

Stoe IPDSII diffractometer
Absorption correction: numerical [shape of crystal determined optically (*X-SHAPE* and *X-RED*; Stoe & Cie, 2005 ▶)] *T*_min_ = 0.980, *T*_max_ = 0.990
15596 measured reflections
2456 independent reflections
2346 reflections with *I* > 2σ(*I*)
*R*_int_ = 0.047

#### Refinement

*R*[*F* ^2^ > 2σ(*F* ^2^)] = 0.031
*wR*(*F* ^2^) = 0.079
*S* = 1.12
2456 reflections
244 parameters
H-atom parameters constrained
Δρ_max_ = 0.21 e Å^−3^
Δρ_min_ = −0.21 e Å^−3^

### 

Data collection: *X-AREA* (Stoe & Cie, 2005 ▶); cell refinement: *X-AREA*; data reduction: *X-RED* (Stoe & Cie, 2005 ▶); program(s) used to solve structure: *SHELXS97* (Sheldrick, 2008 ▶); program(s) used to refine structure: *SHELXL97* (Sheldrick, 2008 ▶); molecular graphics: *X-STEP32* (Stoe & Cie, 2000 ▶); software used to prepare material for publication: *SHELXL97*.

## Supplementary Material

Crystal structure: contains datablocks I, global. DOI: 10.1107/S1600536808003085/hk2424sup1.cif

Structure factors: contains datablocks I. DOI: 10.1107/S1600536808003085/hk2424Isup2.hkl

Additional supplementary materials:  crystallographic information; 3D view; checkCIF report

## Figures and Tables

**Table 1:** Hydrogen-bond geometry (Å, °)

*D*—H⋯*A*	*D*—H	H⋯*A*	*D*⋯*A*	*D*—H⋯*A*
C15—H15*A*⋯O4^i^	0.97	2.40	3.189 (2)	138

Symmetry code: (i) 
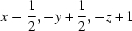
.

**Fig. 1.**
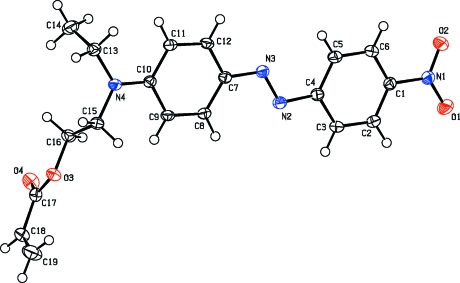
The molecular structure of the title molecule, with the atom-numbering scheme. Displacement ellipsoids are drawn at the 50% probability level.

**Fig. 2.**
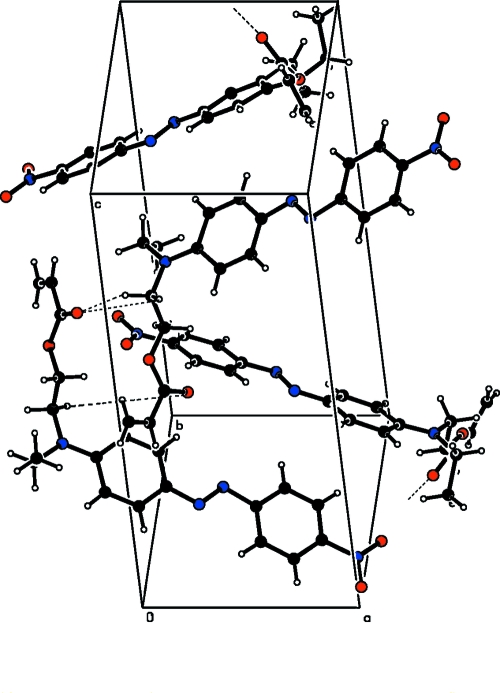
A packing diagram for (I). Hydrogen bonds are shown as dashed lines.

## supplementary crystallographic information

### Comment

It is well known for many years that dyes have been most widely used in fields such as dyeing textile fibers, biomedical studies, advanced applications in organic synthesis and high technology areas like lasers, liquid crystalline displays, electrooptical devices and ink-jet printers (Peters & Freeman, 1991; Gregory 1991; Gur *et al.*, 2007). Azo colorants are the most versatile class of dyes (Venkataraman 1970). They can also be used as indicators in chemical laboratories and as stains in the biological field (Srinivasa *et al.*, 2003). We report herein the synthesis and crystal structure of the title compound, (I).

In the molecule of the title compound, (I), (Fig. 1) the bond lengths and angles are within normal ranges (Lacroix *et al.*, 2000; Gunnlaugsson *et al.*, 2001). Rings A (C1—C6) and B (C7—C12) are, of course, planar and the dihedral angle between them is 0.76 (3)°, so they are also almost coplanar. The atoms N1, N2, N3, N4, O1 and O2 are at the distances of -0.124 (2) Å, 0.070 (3) Å, -0.016 (2) Å, 0.162 (3) Å, -0.066 (3) Å and -0.255 (2) Å, respectively, to the best plane of the coplanar rings.

In the crystal structure, intermolecular C—H···O hydrogen bonds link the molecules, in which they may be effective in the stabilization of the structure.

### Experimental

For the preparation of the title compound, (I), to a magnetically stirred solution of 4-nitro-4'-[*N*-ethyl-*N*-(2-hydroxyethyl)-amino]azobenzene (2.48 mmol) in THF (20 ml), was added dropwise acryloyl chloride (2.48 mmol) in dry nitrogen atmosphere. After 2 h, the mixture was filtered and the desired product was precipitated out by adding water. The solid filtered and washed several times with water, and then dried. The orange precipitated product was recrystallized from ethyl alcohol. After 72 h, orange plate crystals of (I) were isolated (yield; 52.0%, m.p. 397–398 K).

### Refinement

H atoms were positioned geometrically with C—H = 0.93, 0.97 and 0.96 Å for aromatic, methylene and methyl H, respectively, (the two H atoms of atom C19 with C—H = 0.93 Å) and constrained to ride on their parent atoms, with *U*_iso_(H) = 1.2*U*_eq_(C).

### Crystal data

**Table:**

C_19_H_20_N_4_O_4_	*F*_000_ = 776
*M**_r_* = 368.39	*D*_x_ = 1.361 Mg m^−^^3^
Orthorhombic, *P*2_1_2_1_2_1_	Mo *K*α radiation λ = 0.71073 Å
Hall symbol: P 2ac 2ab	Cell parameters from 5000 reflections
*a* = 8.1518 (9) Å	θ = 2.0–27.9º
*b* = 10.6651 (11) Å	µ = 0.10 mm^−^^1^
*c* = 20.6782 (19) Å	*T* = 120 (2) K
*V* = 1797.8 (3) Å^3^	Plate, orange
*Z* = 4	0.5 × 0.2 × 0.06 mm

### Data collection

**Table:**

Stoe IPDSII diffractometer	2456 independent reflections
Radiation source: fine-focus sealed tube	2346 reflections with *I* > 2σ(*I*)
Monochromator: graphite	*R*_int_ = 0.047
Detector resolution: 0.15 mm pixels mm^-1^	θ_max_ = 27.9º
*T* = 120(2) K	θ_min_ = 2.0º
rotation method scans	*h* = −10→10
Absorption correction: numerical[shape of crystal determined optically (X-SHAPE and X-RED; Stoe & Cie, 2005)]	*k* = −14→14
*T*_min_ = 0.980, *T*_max_ = 0.990	*l* = −27→26
15596 measured reflections	

### Refinement

**Table:**

Refinement on *F*^2^	Secondary atom site location: difference Fourier map
Least-squares matrix: full	Hydrogen site location: inferred from neighbouring sites
*R*[*F*^2^ > 2σ(*F*^2^)] = 0.031	H-atom parameters constrained
*wR*(*F*^2^) = 0.079	*w* = 1/[σ^2^(*F*_o_^2^) + (0.041*P*)^2^ + 0.3046*P*] where *P* = (*F*_o_^2^ + 2*F*_c_^2^)/3
*S* = 1.12	(Δ/σ)_max_ = 0.005
2456 reflections	Δρ_max_ = 0.21 e Å^−^^3^
244 parameters	Δρ_min_ = −0.21 e Å^−^^3^
Primary atom site location: structure-invariant direct methods	Extinction correction: none

### Special details

**Table:**

Geometry. All e.s.d.'s (except the e.s.d. in the dihedral angle between two l.s. planes) are estimated using the full covariance matrix. The cell e.s.d.'s are taken into account individually in the estimation of e.s.d.'s in distances, angles and torsion angles; correlations between e.s.d.'s in cell parameters are only used when they are defined by crystal symmetry. An approximate (isotropic) treatment of cell e.s.d.'s is used for estimating e.s.d.'s involving l.s. planes.
Refinement. Refinement of *F*^2^ against ALL reflections. The weighted *R*-factor *wR* and goodness of fit *S* are based on *F*^2^, conventional *R*-factors *R* are based on *F*, with *F* set to zero for negative *F*^2^. The threshold expression of *F*^2^ > σ(*F*^2^) is used only for calculating *R*-factors(gt) *etc*. and is not relevant to the choice of reflections for refinement. *R*-factors based on *F*^2^ are statistically about twice as large as those based on *F*, and *R*- factors based on ALL data will be even larger.

### Fractional atomic coordinates and isotropic or equivalent isotropic displacement parameters (Å^2^)

**Table:**

	*x*	*y*	*z*	*U*_iso_*/*U*_eq_	
O1	1.10305 (16)	0.14200 (13)	0.09457 (7)	0.0364 (3)	
O2	1.03152 (16)	−0.04894 (11)	0.07258 (7)	0.0313 (3)	
O3	−0.36429 (14)	0.34551 (10)	0.47252 (5)	0.0216 (2)	
O4	−0.21122 (16)	0.32212 (12)	0.56227 (6)	0.0294 (3)	
N1	1.00462 (17)	0.05502 (13)	0.09634 (7)	0.0222 (3)	
N2	0.40683 (16)	0.13800 (12)	0.22729 (6)	0.0205 (3)	
N3	0.30983 (16)	0.04473 (12)	0.22891 (6)	0.0192 (3)	
N4	−0.29651 (17)	0.09198 (12)	0.35372 (6)	0.0190 (3)	
C1	0.84626 (18)	0.07543 (14)	0.12848 (7)	0.0189 (3)	
C2	0.8210 (2)	0.18816 (14)	0.16069 (8)	0.0207 (3)	
H2	0.9010	0.2503	0.1607	0.025*	
C3	0.6730 (2)	0.20566 (14)	0.19291 (8)	0.0207 (3)	
H3	0.6533	0.2803	0.2148	0.025*	
C4	0.55405 (18)	0.11203 (14)	0.19253 (7)	0.0188 (3)	
C5	0.5827 (2)	−0.00013 (15)	0.15898 (8)	0.0236 (3)	
H5	0.5026	−0.0622	0.1585	0.028*	
C6	0.7296 (2)	−0.01923 (14)	0.12656 (8)	0.0224 (3)	
H6	0.7493	−0.0934	0.1042	0.027*	
C7	0.16339 (18)	0.06323 (14)	0.26308 (7)	0.0182 (3)	
C8	0.12233 (19)	0.17040 (14)	0.29917 (7)	0.0184 (3)	
H8	0.1962	0.2367	0.3020	0.022*	
C9	−0.02636 (19)	0.17795 (13)	0.33038 (7)	0.0185 (3)	
H9	−0.0505	0.2492	0.3545	0.022*	
C10	−0.14435 (18)	0.07970 (14)	0.32673 (7)	0.0170 (3)	
C11	−0.0979 (2)	−0.03058 (13)	0.29288 (7)	0.0190 (3)	
H11	−0.1685	−0.0990	0.2917	0.023*	
C12	0.05171 (19)	−0.03651 (14)	0.26169 (7)	0.0190 (3)	
H12	0.0791	−0.1089	0.2391	0.023*	
C13	−0.4126 (2)	−0.01280 (14)	0.35654 (8)	0.0218 (3)	
H13A	−0.4112	−0.0565	0.3154	0.026*	
H13B	−0.5223	0.0202	0.3629	0.026*	
C14	−0.3749 (2)	−0.10628 (16)	0.41043 (9)	0.0302 (4)	
H14A	−0.3785	−0.0642	0.4514	0.036*	
H14B	−0.2676	−0.1411	0.4039	0.036*	
H14C	−0.4549	−0.1723	0.4098	0.036*	
C15	−0.35038 (19)	0.21176 (14)	0.38075 (8)	0.0197 (3)	
H15A	−0.4682	0.2195	0.3756	0.024*	
H15B	−0.2990	0.2797	0.3571	0.024*	
C16	−0.3070 (2)	0.22308 (14)	0.45203 (7)	0.0212 (3)	
H16A	−0.3605	0.1575	0.4768	0.025*	
H16B	−0.1894	0.2161	0.4581	0.025*	
C17	−0.3027 (2)	0.38472 (15)	0.52915 (8)	0.0218 (3)	
C18	−0.3577 (2)	0.51211 (16)	0.54784 (8)	0.0263 (3)	
H18	−0.3365	0.5385	0.5899	0.032*	
C19	−0.4339 (3)	0.58999 (18)	0.50938 (10)	0.0398 (5)	
H19A	−0.4570	0.5667	0.4670	0.048*	
H19B	−0.4649	0.6687	0.5244	0.048*	

### Atomic displacement parameters (Å^2^)

**Table:**

	*U*^11^	*U*^22^	*U*^33^	*U*^12^	*U*^13^	*U*^23^
O1	0.0293 (6)	0.0269 (6)	0.0529 (8)	−0.0081 (5)	0.0145 (6)	−0.0026 (6)
O2	0.0316 (6)	0.0222 (5)	0.0401 (7)	0.0030 (5)	0.0113 (6)	−0.0058 (5)
O3	0.0232 (5)	0.0183 (5)	0.0235 (5)	0.0039 (4)	−0.0010 (4)	−0.0038 (4)
O4	0.0289 (6)	0.0331 (6)	0.0261 (6)	0.0055 (6)	−0.0039 (5)	−0.0022 (5)
N1	0.0223 (6)	0.0194 (6)	0.0250 (7)	0.0007 (5)	0.0034 (5)	0.0016 (5)
N2	0.0205 (6)	0.0176 (6)	0.0233 (6)	−0.0009 (5)	0.0001 (5)	−0.0010 (5)
N3	0.0196 (6)	0.0169 (6)	0.0211 (6)	−0.0001 (5)	0.0006 (5)	0.0007 (5)
N4	0.0204 (6)	0.0143 (5)	0.0224 (6)	0.0011 (5)	0.0014 (5)	−0.0009 (5)
C1	0.0198 (7)	0.0177 (7)	0.0193 (7)	0.0007 (6)	0.0014 (6)	0.0012 (6)
C2	0.0211 (7)	0.0161 (6)	0.0248 (7)	−0.0023 (6)	−0.0005 (6)	0.0008 (6)
C3	0.0235 (8)	0.0145 (6)	0.0240 (7)	−0.0001 (6)	−0.0003 (6)	−0.0021 (6)
C4	0.0187 (7)	0.0179 (7)	0.0198 (6)	0.0003 (6)	−0.0006 (6)	0.0005 (6)
C5	0.0242 (7)	0.0185 (7)	0.0281 (8)	−0.0051 (6)	0.0029 (6)	−0.0048 (6)
C6	0.0256 (8)	0.0165 (7)	0.0252 (7)	−0.0015 (6)	0.0029 (6)	−0.0038 (6)
C7	0.0189 (7)	0.0161 (7)	0.0195 (7)	0.0015 (6)	−0.0007 (6)	0.0008 (5)
C8	0.0208 (7)	0.0138 (6)	0.0206 (6)	−0.0004 (6)	−0.0007 (6)	0.0005 (6)
C9	0.0233 (7)	0.0124 (6)	0.0196 (6)	0.0010 (6)	−0.0005 (6)	−0.0006 (5)
C10	0.0199 (7)	0.0146 (6)	0.0166 (6)	0.0018 (5)	−0.0014 (5)	0.0018 (5)
C11	0.0217 (7)	0.0144 (6)	0.0208 (7)	−0.0014 (6)	−0.0008 (6)	−0.0008 (6)
C12	0.0232 (7)	0.0129 (6)	0.0210 (7)	0.0025 (6)	−0.0001 (6)	−0.0019 (6)
C13	0.0197 (7)	0.0199 (7)	0.0258 (7)	−0.0021 (6)	0.0009 (6)	−0.0009 (6)
C14	0.0395 (9)	0.0207 (7)	0.0303 (8)	−0.0048 (7)	0.0005 (8)	0.0047 (7)
C15	0.0205 (7)	0.0154 (6)	0.0233 (7)	0.0049 (6)	0.0012 (6)	0.0009 (6)
C16	0.0247 (7)	0.0167 (6)	0.0221 (7)	0.0042 (6)	0.0005 (6)	−0.0005 (6)
C17	0.0208 (7)	0.0231 (7)	0.0214 (7)	−0.0010 (6)	0.0037 (6)	−0.0002 (6)
C18	0.0306 (8)	0.0232 (7)	0.0251 (7)	−0.0011 (7)	0.0025 (7)	−0.0063 (6)
C19	0.0603 (13)	0.0257 (8)	0.0333 (9)	0.0090 (9)	−0.0009 (9)	−0.0063 (8)

### Geometric parameters (Å, °)

**Table:**

C1—C6	1.388 (2)	C13—N4	1.466 (2)
C1—C2	1.390 (2)	C13—C14	1.527 (2)
C1—N1	1.4681 (19)	C13—H13A	0.9700
C2—C3	1.391 (2)	C13—H13B	0.9700
C2—H2	0.9300	C14—H14A	0.9600
C3—C4	1.392 (2)	C14—H14B	0.9600
C3—H3	0.9300	C14—H14C	0.9600
C4—C5	1.402 (2)	C15—N4	1.4618 (18)
C4—N2	1.4261 (19)	C15—C16	1.520 (2)
C5—C6	1.387 (2)	C15—H15A	0.9700
C5—H5	0.9300	C15—H15B	0.9700
C6—H6	0.9300	C16—O3	1.4500 (17)
C7—C12	1.400 (2)	C16—H16A	0.9700
C7—N3	1.4012 (19)	C16—H16B	0.9700
C7—C8	1.406 (2)	C17—O4	1.213 (2)
C8—C9	1.376 (2)	C17—O3	1.3412 (19)
C8—H8	0.9300	C17—C18	1.482 (2)
C9—C10	1.424 (2)	C18—C19	1.307 (3)
C9—H9	0.9300	C18—H18	0.9300
C10—N4	1.367 (2)	C19—H19A	0.9300
C10—C11	1.420 (2)	C19—H19B	0.9300
C11—C12	1.381 (2)	N1—O1	1.2271 (18)
C11—H11	0.9300	N1—O2	1.2323 (18)
C12—H12	0.9300	N2—N3	1.2712 (18)
			
C6—C1—C2	122.79 (14)	N4—C13—H13B	108.9
C6—C1—N1	118.80 (13)	C14—C13—H13B	108.9
C2—C1—N1	118.39 (13)	H13A—C13—H13B	107.7
C1—C2—C3	118.30 (14)	C13—C14—H14A	109.5
C1—C2—H2	120.8	C13—C14—H14B	109.5
C3—C2—H2	120.8	H14A—C14—H14B	109.5
C2—C3—C4	120.35 (14)	C13—C14—H14C	109.5
C2—C3—H3	119.8	H14A—C14—H14C	109.5
C4—C3—H3	119.8	H14B—C14—H14C	109.5
C3—C4—C5	119.92 (14)	N4—C15—C16	111.73 (13)
C3—C4—N2	116.35 (13)	N4—C15—H15A	109.3
C5—C4—N2	123.73 (14)	C16—C15—H15A	109.3
C6—C5—C4	120.54 (15)	N4—C15—H15B	109.3
C6—C5—H5	119.7	C16—C15—H15B	109.3
C4—C5—H5	119.7	H15A—C15—H15B	107.9
C5—C6—C1	118.09 (14)	O3—C16—C15	106.25 (12)
C5—C6—H6	121.0	O3—C16—H16A	110.5
C1—C6—H6	121.0	C15—C16—H16A	110.5
C12—C7—N3	115.89 (13)	O3—C16—H16B	110.5
C12—C7—C8	118.28 (13)	C15—C16—H16B	110.5
N3—C7—C8	125.81 (14)	H16A—C16—H16B	108.7
C9—C8—C7	120.43 (14)	O4—C17—O3	123.49 (15)
C9—C8—H8	119.8	O4—C17—C18	122.93 (15)
C7—C8—H8	119.8	O3—C17—C18	113.59 (14)
C8—C9—C10	121.81 (13)	C19—C18—C17	124.60 (16)
C8—C9—H9	119.1	C19—C18—H18	117.7
C10—C9—H9	119.1	C17—C18—H18	117.7
N4—C10—C11	121.51 (13)	C18—C19—H19A	120.0
N4—C10—C9	121.39 (13)	C18—C19—H19B	120.0
C11—C10—C9	117.09 (13)	H19A—C19—H19B	120.0
C12—C11—C10	120.23 (14)	O1—N1—O2	123.50 (13)
C12—C11—H11	119.9	O1—N1—C1	118.44 (13)
C10—C11—H11	119.9	O2—N1—C1	118.05 (13)
C11—C12—C7	121.99 (14)	N3—N2—C4	112.63 (12)
C11—C12—H12	119.0	N2—N3—C7	115.66 (12)
C7—C12—H12	119.0	C10—N4—C15	120.84 (13)
N4—C13—C14	113.39 (14)	C10—N4—C13	121.96 (12)
N4—C13—H13A	108.9	C15—N4—C13	117.20 (13)
C14—C13—H13A	108.9	C17—O3—C16	114.54 (12)
			
C6—C1—C2—C3	0.8 (2)	O3—C17—C18—C19	−11.8 (3)
N1—C1—C2—C3	−177.92 (13)	C6—C1—N1—O1	175.31 (15)
C1—C2—C3—C4	0.0 (2)	C2—C1—N1—O1	−6.0 (2)
C2—C3—C4—C5	−0.6 (2)	C6—C1—N1—O2	−4.8 (2)
C2—C3—C4—N2	179.52 (14)	C2—C1—N1—O2	173.98 (15)
C3—C4—C5—C6	0.6 (2)	C3—C4—N2—N3	−174.90 (13)
N2—C4—C5—C6	−179.55 (15)	C5—C4—N2—N3	5.3 (2)
C4—C5—C6—C1	0.1 (2)	C4—N2—N3—C7	179.20 (12)
C2—C1—C6—C5	−0.8 (2)	C12—C7—N3—N2	176.24 (13)
N1—C1—C6—C5	177.89 (14)	C8—C7—N3—N2	−5.4 (2)
C12—C7—C8—C9	−2.1 (2)	C11—C10—N4—C15	−171.70 (14)
N3—C7—C8—C9	179.54 (14)	C9—C10—N4—C15	7.2 (2)
C7—C8—C9—C10	−0.9 (2)	C11—C10—N4—C13	7.9 (2)
C8—C9—C10—N4	−174.91 (14)	C9—C10—N4—C13	−173.24 (14)
C8—C9—C10—C11	4.0 (2)	C16—C15—N4—C10	−90.19 (16)
N4—C10—C11—C12	174.82 (13)	C16—C15—N4—C13	90.21 (17)
C9—C10—C11—C12	−4.1 (2)	C14—C13—N4—C10	78.35 (18)
C10—C11—C12—C7	1.2 (2)	C14—C13—N4—C15	−102.06 (16)
N3—C7—C12—C11	−179.50 (14)	O4—C17—O3—C16	−2.6 (2)
C8—C7—C12—C11	2.0 (2)	C18—C17—O3—C16	177.69 (13)
N4—C15—C16—O3	179.17 (12)	C15—C16—O3—C17	−164.62 (13)
O4—C17—C18—C19	168.5 (2)		

### Hydrogen-bond geometry (Å, °)

**Table:**

*D*—H···*A*	*D*—H	H···*A*	*D*···*A*	*D*—H···*A*
C15—H15A···O4^i^	0.97	2.40	3.189 (2)	138

Symmetry codes: (i) *x*−1/2, −*y*+1/2, −*z*+1.
